# Concentration–Response Functions as an Essence of the Results from Lags

**DOI:** 10.3390/ijerph19138116

**Published:** 2022-07-01

**Authors:** Mieczysław Szyszkowicz

**Affiliations:** Environmental Health Science and Research Bureau, Health Canada, Ottawa, ON K1A 0K9, Canada; mszyszkowicz@yahoo.ca

**Keywords:** air pollution, case-crossover, concentration, ozone, respiratory problems, response

## Abstract

Among various aspects of environmental epidemiology, one is to assess the relationships between ambient air pollution and health outcomes. The goal of this work is to estimate the associations in the form of the parametric concentration–response functions (C-RF). Various forms of the C-RFs are proposed in this short-term health effect study. Emergency department (ED) visits for all respiratory health problems are analyzed as an illustrative example. A case-crossover (CC) technique is applied as a study design. Daily cases are organized as daily counts by the same day of the week in one common month. A conditional Poisson regression is used in the constructed statistical models. Temperature and relative humidity are included in the statistical models in the form of natural splines. Ground-level ozone concentration is considered an exposure. Ozone concentration values are transformed and submitted to the statistical models. The parameters of the transformation are determined by using the goodness of fit criterion. Counts of ED visits are analyzed in relation to a sequence of lagged exposure to ozone. The C-RF shapes are constructed for each individual lag. In a final step, the set of the estimated C-RF shapes is used to create a pooled C-RF shape. The results are positive and statistically significant for nine lagged exposures, from 0 to 8 days. The following relative risks (RR) were estimated from the constructed C-RFs at 30 ppb concentration of ozone: RR = 1.0531 (95% confidence interval: 1.0231, 1.0718), 1.0462 (1.0253, 1.0677), and 1.0387, (1.0240, 1.0531), realizing the CC method, CC method + transformation, and CC method + flexible transformation, respectively. The pooled C-RF shape gives a summary of the associations between ED visits for respiratory conditions and ambient ozone. The estimated shapes indicate lower air health effects than the standard CC methods. Among three considered statistical models, the CC method + flexible transformation is the most appropriate to use according to the goodness of fit criterion.

## 1. Introduction

In this paper, a technique to estimate the associations between the concentration levels of ambient air pollution and health outcomes is presented. The described technique is realized in a short-term air health effect scenario. The main goal of this work is to estimate the association in the form of a parametric algebraic function, which is the concentration–response function (C-RF). The C-RF shape determines the health risk as a function of the air pollutant concentration levels. These shapes are important tools in estimating air pollution health effects [1,2,3,4,5]. The CR-F profile provides knowledge of both the shape (profile) and magnitude of the relative risk along the concentration values. The C-RF shapes allow for the determination of a potential threshold. The ranges of air pollution concentration are studied in the short-term exposure effects. Daily counts of health data and daily air pollutant concentration levels are used in this study. In addition, two weather factors, temperature and relative humidity, are included in the constructed statistical models. The air health effect of exposure to air pollutants is analyzed for a series of daily lags. In the literature, the results from these types of studies are usually obtained for individually considered lags [6,7]. Traditionally, associations are reported for the lags, which indicate the largest estimated health effects among the considered lagged exposure. Another approach to analyzing the impact of the lagged exposures is to show the associations between the realized lags by applying distributed lag methods [8,9].

The traditional approach to estimating risk related to short-term health effects is to use a time-series analysis (based on counts) or case-crossover (based on separate health events). Usually, two kinds of functions are used to represent the relative risk as a function of concentration. One is an exponential function with the coefficients estimated on a log-linear scale, and another is to use the splines to adjust for the fluctuations related to air pollution concentrations. Here, other kinds of relationships are described.

This work proposes a technique that merges the estimated C-RF shapes constructed for the individual lags. The C-RF shapes are usually different in their forms by the applied individual lags. These C-RF shapes are well-described by their parameters. The used statistical models allow for the estimation of an optimal set of parameters. The applied fit criteria provide a measure of goodness of fit for the constructed statistical models. An optimal set of parameters is chosen using a measure of goodness of fit. In this work, the Akaike information criterion (AIC) is used to classify the generated estimations [10,11].

The results of the associations are reported as the concentration–response function (C-RF) for each individually considered lag. The next step in the proposed methodology is to execute a kind of meta-analysis using the constructed algebraic functions and to obtain a pooled single risk estimate value for all lag days. In this work, the estimation of a global concentration–response function, which summarizes the results, is proposed. As a result, a single concentration–response function is built based on a series of these functions produced for the individually considered lags.

The main contribution of this paper is to construct an algebraic C-RF, which summarizes the results generated for a series of lagged exposure. The main motivation of the paper is to present a tool for policymakers to assess the risks for various air pollutant concentrations.

Constructing the concentration–response relationship of air pollutant levels and health conditions is very important and often a challenging problem. One of the problems is to detect the presence of a potential threshold, such that air pollutant levels below this threshold are not expected to generate adverse health effects. Another problem is to prevent the responses when the estimated risk is decreasing for increasing concentration levels. This is mainly due to the situation that higher levels than “normal” ones are relatively rare. As a consequence, the air health effects are not reflected properly. In the presented study, a very high concentration of ambient ozone does not necessarily increase the number of ED visits for respiratory conditions. These situations are not very common, but they produce a decreasing risk. This is usually observed when in the study splines are used to model the C-RFs. The technique presented here resolves these two problems; it identifies a threshold, and the constructed C-RF is always a non-decreasing monotiling function.

## 2. Materials and Methods

### 2.1. Health Data

The health data are values related to emergency department (ED) visits in Edmonton, Canada, in the period from April 1992 to March 2002. The ED visit data were collected from five different hospitals in the greater Edmonton region over a 10-year period. These data are implemented here for an illustrative purpose. The time period consists of a total of 3652 consecutive days. All ED visits diagnosed as diseases of the respiratory system (International Classification of Diseases, ICD-9; classified by the ICD-9 codes: 460–519) were considered the same with one common health problem. For the presented technique, the data were organized as daily counts of the ED visits. Among over 2 million records stored in the used health database, 292,285 ED visits were identified, retrieved, and used in the study.

An air pollutant ambient ozone (O_3_) concentration was considered. Its levels were estimated and represented as an 8 h daily maximum concentration. Daily average ambient temperature and relative humidity were applied to represent these weather values. The weather parameters in the constructed models were included in the form of natural spines. The environmental data were retrieved from the National Air Pollution Surveillance (NAPS) database, maintained by Environment and Climate Change Canada [12].

### 2.2. Statistical Model

Let Z denote daily air pollution concentration levels. In the present study, Z = O_3_, where ambient ozone is measured as a maximum of averaged concentrations over an 8 h period. The constructed models are realized by implementing a conditional Poisson regression. The conditional Poisson model is conditioning on the total event count in each stratum. The time stratified case-crossover (CC) technique is applied to determine the strata, which are composed of 4 or 5 days [13,14]. The constructed strata match days are based on a hierarchical calendar structure, which is as follows: same day of the week, calendar month, and year. These constructions have previously been used to minimize bias among various approaches to define control periods for the case event in the case-crossover models [13]. This construction can eliminate time-invariant factors such as sex, smoking, and socioeconomic position. It has been shown that a conditional Poisson regression model gives equivalent estimates as the conditional logistic regression model [15]. The models realized here allow adjusting for over-dispersion (using the option: quasi-Poisson). In this case, there is no AIC value.

The models are implemented in the R statistical software (R Foundation for Statistical Computing, Vienna, Austriay) [16] and have the following form:

*ModelFit* = *gnm* (*Health* ~ *AP(L)* + *ns(Temp(L)*,3) + *ns(RHum(L)*,3), *data* =* data, family* = *Poisson, eliminate* = *factor(stratum)*)

where *AP*, *Temp*, and *RHum* are values of the used air pollutant (ozone), temperature, and relative humidity, respectively. All these three factors are lagged by the same number of days, as marked here by the letter *L* (Lag). The used stratum indicator has the form <*year:month:day-of-week*>. The variable *Health* represents daily counts of the considered health conditions. In the presented situation, these are daily ED visits for respiratory problems. The *gnm* package fits generalized non-linear models [16,17]. The estimated coefficient for *AP*, called *Beta* (slope), with its standard error (*SE*), is used to calculate relative risk (*RR*) and its 95% confidence interval (95%CI).

### 2.3. Transformation

The statistical models are built for various transformations of the *AP* represented by the variable *Z.* The models are constructed using *AP* in the form *AP = T*(*Z*), where *T*(*Z*) is the transformation of *Z*. The following techniques are proposed and conducted.

- No transformation; $AP=T\left(Z\right)=Z,\;\mathrm{and}\;RR\left(Z\right)=exp\left(Beta\times Z\right)$. It is a classical approach on the log-linear scale. Here, it is called the CC method.
- The transformation has the form $T\left(Z\right)=f\left(Z\right)\times LWF\left(Z\right)$, where *f(Z)* is a simple function of *Z*, such as *Z*, *log(Z)*, *sqrt(Z)* (= $\sqrt{Z})$, or some other powers of *Z* ($f\left(Z\right)=Z^{P}),$ with *P* greater or lower than 1, i.e., convex and concave functions of the variable *Z*, respectively.
- The transformation has the following form $T\left(Z\right)=f\left(Z\right)\times LWF\left(Z\right)$, with the function $f\left(Z\right)=log\left(1+Z/A\right)$, where *A* is a parameter. This form is also used to represent a pooled C-RF shape.

In the above notation, *LWF* represents a logistic weighting function. This function is described by the following formula:

$$LWF\left(Z\right)=\frac{1}{1+exp\left\{\frac{\mu -Z}{r*\tau }\right\}}\;,\;$$

where *mu* and *tau* (μ,τ) are the parameters, and *r* is the concentration level range, i.e., the range of *Z* measured in the study. The parameters μ,τ, and A (*mu*, *tau*, and *A*), for a given initial guess, are determined by an iterative algorithm, which minimizes the AIC value for the fitted models. Another approach to determine an optimal transformation is to tabulate the transformation functions with various values of its parameters. It is a reasonable method to realize the variant (b) presented above. In this situation, the values of *mu* are taken as percentiles of *Z*, and *tau* is tested for two values, 0.1 and 0.2 [18]. The coefficient *Beta* and its standard error are estimated by the realized statistical models applied to fit the health data.

The classical CC technique traditionally calculates the relative risk as $RR\left(Z\right)=exp\left(Beta\times Z\right)$. The present approach gives the risk as $RR\left(Z\right)=exp\left(Beta\times T\left(Z\right)\right)$. The logistic function used in the transformation, *LWF(Z*), allows adapting to various shapes of the concentration–response. The proposed method usually gives the results with a better fit than the standard CC method, i.e., with no-transformation *T*(*Z*). The presented technique estimates the best model according to the criteria used and provides the coefficient *Beta* and its standard error (*SE*).

The next step is to summarize the generated C-RF shapes by a sequence of the used lags. The relative risk *RR* at a point *Z* is represented as $RR\left(Z\right)=exp\left(\beta \left(Z\right)\right)$, where

$$\beta \left(Z\right)=\theta \times log\left(1+Z/A\right)\times LWF\left(Z,\tau ,\mu \right).\;$$

The final C-RF shape has the following form (*RR*(*Z*))

$$RR\left(Z\right):{\left(1+Z/A\right)}^{\theta *LWF}\;$$

In this step, the parameters A, τ,μ, and θ {*A*, *tau*, *mu*, and *Theta*} are estimated using a least-square approximation. The function C−RF is fitted to the series of C-RF shapes generated for the individual lags.

In addition, knowing the standard error of the estimated *Betas*, the set of lower and upper values of the C-RF shapes are produced and fitted with the function of the same forms. Thus, the upper 95% confidence interval limits (a set of C-RFs) and corresponding lower limits (a set of C-RFs) are used to construct two limits. One is based on the upper and another on the lower set of profiles. These two C-RF shapes provide some boundaries for the C-RF shape.

At the following location, https://github.com/szyszkowiczm/FitCRF, (accessed on 28 June 2022) in the form of a pdf file called ProgramToFitCRF, two programs in R are presented. Program 1 fits CR-F for individual lags. Program 2 generates a common CR-F using the results from the applied lagged concentrations. These programs allow the better presentation and interpretation of the proposed methodology. It should be noted that a set of non-linear equations are resolved to determine the constructed C-RF. Moreover, the C-RFs are well-described by the estimated parameters {*A*, *tau*, *mu*, and *Theta*}.

## 3. Results

Table 1 presents the results for ED visits for respiratory conditions in Edmonton, Canada, 1992–2002. The table has three parts, which correspond to the above method description of three approaches (a) *Z* is not transformed, and as a consequence, the standard CC method is realized, (b) *Z* is transformed using the simple function *f*(*Z*) and *LWF*(*Z*), and (c) *Z* is transformed using *f*(*Z*) of the form *log*(1 + *Z*/*A*) and *LWF*(*Z*). The calculations were performed by lags from 0 to 9.

The results for lag 9 were negative (*Beta* < 0). Thus, the C-RF shapes estimated for lagged exposure from 0 to 8 were used to construct a pooled shape as a sort of summary. Table 2 shows the obtained parameters for all three forms of the considered transformations.

For an illustrative purpose, the relative risks were calculated at point *Z* = ozone = 30 ppb. The C-RF shapes defined in Table 2 give the following estimations: (a) *RR* = 1.0531 [1.0231, 1.0718], (b) *RR* = 1.0462 [1.0253, 1.0677], and (c) *RR* = 1.0387 [1.0240, 1.0531]. These values correspond to fit all results (lag 0 to lag 8) with the C-RF shape of the form $RR\left(Z\right)=exp\left(\beta \left(Z\right)\right)$ where $\beta \left(Z\right)=\theta \times log\left(1+Z/A\right)\times LWF\left(Z,\tau ,\mu \right).$ The listed intervals (say [1.0240, 1.0531]) are based on the same fitted type of function as the set of lower and upper boundaries of the 95% confidence intervals. Table 3 shows the assessed RRs at *Z* = 30 ppb for lag 0 estimated by the methods (a)–(c) (given in Table 1) and pooled C-RF shapes (given in Table 2).

**Figure 1:**
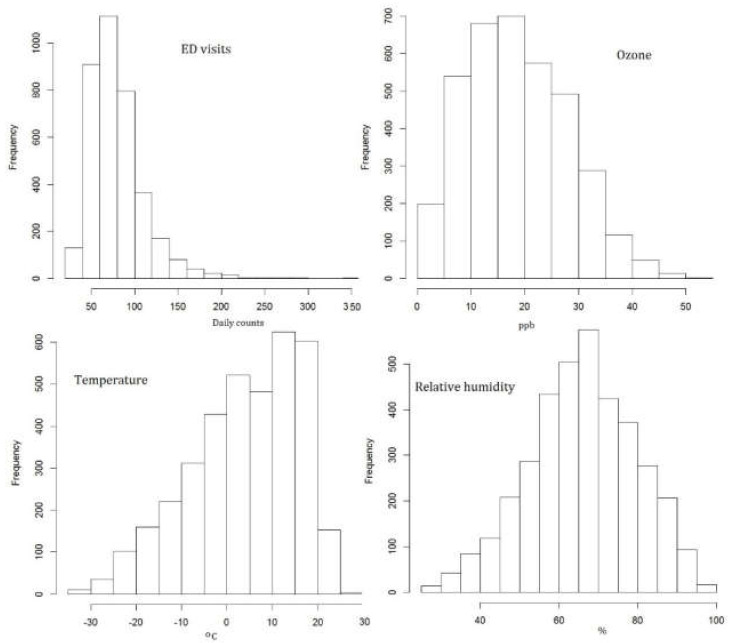
Daily values during the study period (3652 days). Histogram of ED visits (daily counts), ozone (ppb), temperature (°C), and relative humidity (%).

Figure 1 presents the histograms of the considered factors in the analysis; health responses—daily ED visits, ozone, temperature, and relative humidity. The histogram for ozone is mainly interesting in relation to the estimated C-RF shapes. It illustrates the frequencies of various daily concentrations and allows for estimating the intensity of occurring levels with effects on health conditions.

**Figure 2:**
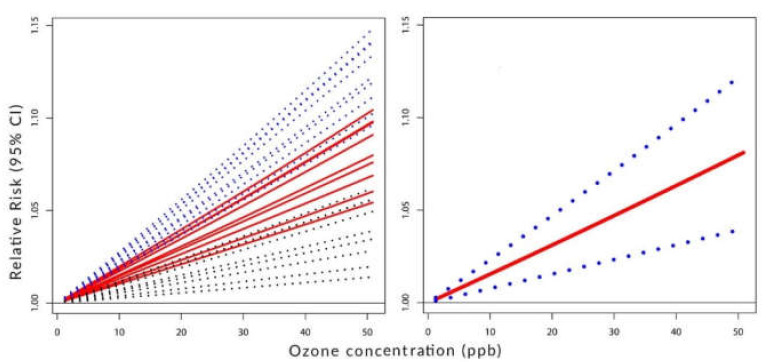
**Left panel**: C-RF shapes by lags. **Right panel**: a pooled C-RF shape. No transformation $\left(f\left(Z\right)=Z\right)$.

**Figure 3:**
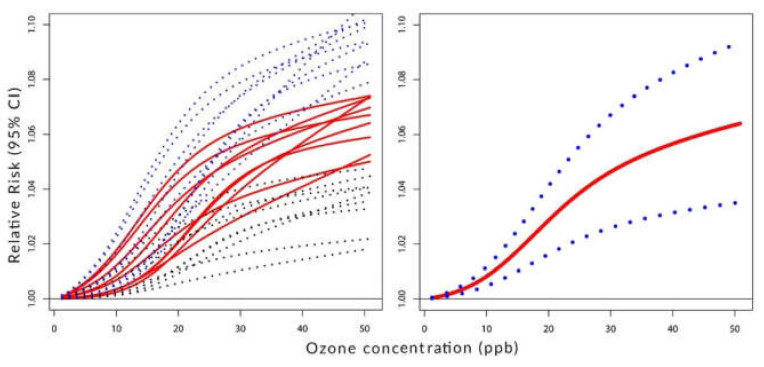
**Left panel**: C-RF shapes by lags. **Right panel**: a pooled C-RF shape. Transformation with *f(Z)* chosen among three simple functions.

**Figure 4:**
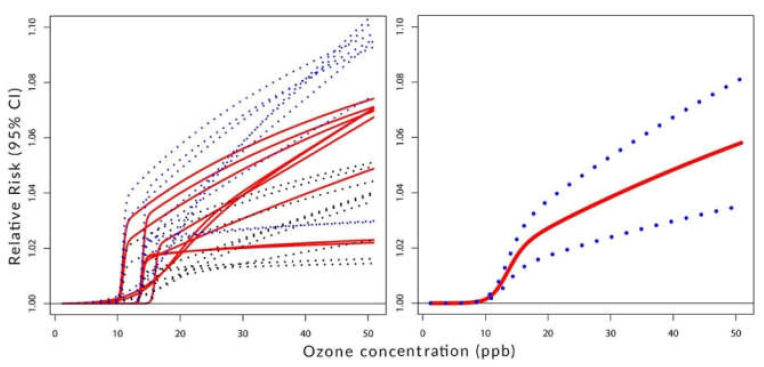
**Left panel**: C-RF shapes by lags. **Right panel**: a pooled C-RF shape. Transformation with $f\left(Z\right)=log\left(1+Z/A\right)$.

Figure 2, Figure 3 and Figure 4 visualize the situation presented numerically in Table 1 and Table 2. Their left panels show the individual components of the constructed C-RF shapes for lags from 0 to 8. Their right panels show the constructed summary, a pooled C-RF shape. To be clear, these C-RF shapes are $RR\left(Z\right)=exp\left(\beta \left(Z\right)\right)$. The lower and upper boundaries are also shown. The figures illustrate the results for the transformation as follows: Figure 2 for (a), Figure 3 for (b), and Figure 4 for (c).

Figure 4 indicates the presence of the threshold in the considered range of concentrations. The ambient ozone with levels below 10 ppb does not show any health effects on human respiratory health conditions.

## 4. Discussion

Exposure to ozone in ambient air has been associated with various health outcomes, but mainly with health problems diagnosed as respiratory conditions. This study supports these associations and indicates a relatively long period of the relations measured by used lags. The positive associations are observed for lagged exposure from 0 to 8 days.

The constructed C-RFs in their forms are algebraic parametric functions. For the positive coefficient (*Beta*) for air pollutants, the constructed C-RFs are non-decreasing monotonic functions. It implies that for higher concentration levels, the estimated RR is not lower than for lower levels. The risk does not decrease as the concentration increases.

The fitted C-RFs are considered in two aspects. One of them is their risk estimate values at a specific O_3_ concentration. Another is their profile and shape. The estimated values of RR have almost the same range for the three proposed approaches (a–c). The main differences are in their shape. The methods, which have a better fit to the data, using the goodness of fit criterion, give the lowest estimation at the point of 30 ppb (see Table 3, AIC values). Moreover, this method suggests the presence of a threshold.

To the best of my knowledge, this is the first study in the literature to use such methodology for estimating the health risks of short-term exposure to air pollutants, mainly to provide a summary risk estimate from all lagged exposure concentration–response functions. The method proposed here collects the results not from various centers but from the same study area using the results obtained for a series of lags. The presented C-RFs indicate that the methodology is reliable. The estimated RR values are very similar in their magnitudes. A least-square method does not include variability and correlations among the generated C-RFs. Another approach is to use the technique presented by other authors [18,19,20]. In this case, the applied methodology is realized in cohort studies (longitudinal studies) using Cox regression. The parameters of the transformations are determined by applying their tabulated values. The algorithm used in [18] to determine the parameters of the weighing functions is as follows: “Step 1. Create four weighting variables based on values of μ defined at the 0th, 25th, 50th, and 75th percentiles of the air pollution distribution with τ = 0.1 and an additional four variables with τ = 0.2.” To have more accurate values of the location parameter (μ), additional steps are realized: “Given the best fitting μ value based on Step 1, fit two models setting μ to five percentile values greater than and less than the best fitting μ. For μ equal to the minimum concentration, subtract and increment equal to the difference between the 5th percentile and minimum concentration from the minimum concentration and denote this value as −5th percentile. Continue to take differences of minimum—10% of increment and minimum—15% of increment until log-likelihood is maximized.”—see the details in [18]. In contrast, here, the parameters of the weighting functions are found by applying a minimization function (not the tabulation as in [18]. In this approach [18], the proposed parameters are submitted to the used statistical models. A set of probable values are tried in a series of models. The used criterion to accept a specific set is the log-likelihood value [18]. In contrast, in this presentation, the parameters of the models are determined by resolving non-linear equations. The technique realized to construct an ensemble model (presented in [19], used in [20]) based on the models derived in various centers can be shortly characterized as [19]: “An ensemble estimate is then constructed of all of the shapes examined weighted by their respective likelihood values. Bootstrap methods were used to obtain uncertainty intervals.” Here, the ensemble model is generated by using the models for individual lags. The practical details are given in the form of the included computer programs (see file ProgramToFitCRF [16,17]).

The approach presented here (case-crossover with the transformation) was also tested for ozone and mortality in Toronto [21,22]. The estimated relative risks (*Z* = 55 μg/m^3^) were *RR* = 1.01947 (95%CI: 1.00295, 1.03627) using the standard case-crossover model, and *RR* = 1.00391 (95%CI: 1.00181, 1.00601) using the case-crossover model with the transformation. The corresponding AIC values were 78,707.7 vs. 78,699.3 [22].

In the presented work, short-term air health effects are analyzed. The presented methodology allows for the determination of a threshold. It is conducted without any special construction of the concentration–response function. Here, air pollutant concentrations are transformed, but the realized statistical method (case-crossover) is standard. This applied transformation results in the C-RF, which is a non-decreasing function of the air pollutant concentration levels.

## 5. Conclusions

It is often observed that epidemiological studies related to health conditions and short-term exposure usually do not detect a threshold. As Figure 2 in this work shows, it may be related to the form of the C-RF shape. In the standard CC method, RR has the form *exp*(*Beta* × *Dose*). These functions do not allow for the identification of a threshold. Using splines to construct the C-RF profiles often results in decreasing risks for increasing concentrations. The proposed here methodology prevents such situations.

As indicated in Nasari et al. [18] (their Figure 1), the form proposed here of the C-RF allows the construction of a variety of shapes from the model specification. These shapes are flexible, and they resolve the problem of the threshold and decrease the risk of higher air pollutant concentration levels.

The present approach allows unifying the forms of relative risks represented as the C-RF profiles constructed for various lags. Using the proposed methods allows detection of a potential threshold. The results demonstrate that the best approach among the considered approaches is the CC method with flexible transformation.

## Figures and Tables

**Table 1:** The estimations obtained by the case-crossover approach: (**a**) no transformation, (**b**) transformation with *f*(*Z*) (*=Z*, *log*(*Z*), or *sqrt*(*Z*)), and (**c**) transformation with *f*(*Z*) *= log*(1 + *Z*/*A*). The transformation is submitted into the models in the form *f*(*Z*) × *LWF*(*Z*) to represent concentrations. ED visits for all respiratory conditions. Edmonton, Canada, 1992–2002.

(a) *RR*(*Z*) = *exp*(*Beta* × *Z*)					
*Lag*	*Beta*	*SE*	*Lag*	*Beta*	*SE*
0	0.00183	0.00039	5	0.00131	0.00039
1	0.00115	0.00039	6	0.00151	0.00039
2	0.00104	0.00039	7	0.00171	0.00039
3	0.00184	0.00039	8	0.00144	0.00039
4	0.00195	0.00039	9	−0.00004	0.00039
**(b) *RR*(*Z*) = *exp*(*β*(*Z*)), *β*(*Z*) = *Beta* × *f*(*Z*) × *LWF*(*Z*,*τ*,*μ*), *τ* = 0.1**					
***Lag***	***Beta***	***SE***	** % **	***μ***	***f(Z)***
0	0.0099	0.0019	39	15.0	sqrt
1	0.0069	0.0020	34	13.8	sqrt
2	0.0010	0.0003	31	13.0	z
3	0.0165	0.0032	26	11.6	log
4	0.0182	0.0032	26	11.6	log
5	0.0146	0.0033	60	20.6	log
6	0.0087	0.0019	57	19.6	sqrt
7	0.0095	0.0020	27	12.0	sqrt
8	0.0014	0.0003	49	17.6	z
9	−0.0041	0.0041	0	1.2	log
**(c) *RR*(*Z*) = *exp*(*β*(*Z*)), *β*(*Z*) = *Beta* × *log*(1 + *Z*/*A*) × *LWF*(*Z*,*τ*,*μ*)**					
***Lag***	***Beta***	***SE***	***μ***	***τ***	***A***
0	0.0467	0.0070	14.0	0.005	15.2
1	0.0035	0.0006	14.0	0.005	0.1
2	0.1220	0.0325	13.8	0.005	106.5
3	0.0047	0.0007	13.9	0.005	0.4
4	0.0414	0.0064	10.8	0.005	11.0
5	7.3226	1.4687	16.0	0.005	5690.1
6	4.3945	0.9644	17.4	0.061	3250.2
7	0.0650	0.0118	10.9	0.005	28.0
8	326.6490	77.3902	17.4	0.081	243,940.0
9	−0.0085	0.0018	3.1	0.005	0.0

**Table 2:** Estimations based on the RR values calculated for lag from 0 to 8. Three forms of the input (**a**)–(**c**) are realized. ED visits for all respiratory conditions. Edmonton, Canada, 1992–2002.

Parameters	Value	*SE*	Low Value	*SE*	Upper Value	*SE*
Output	(a) Input: *RR*(*Z*) = *exp*(*Beta* × Z)					
θ	0.4	4.5	2.9	5052.0	2.3	1715.4
A	130.7	1805.4	3275.0	6,745,000.0	748.9	745,095.2
μ	−27.6	655.0	−1158.0	822,200.0	−388.6	415,055.2
τ	2.3	27.6	12.9	7879.0	7.0	1336.5
**Output**	**(b) Input: *RR*(*Z*) = *exp*(*β*(*Z*)),*β* = *Beta* × *f*(*Z*) × *LWF*(*Z*,*τ*,*μ*)**					
θ	0.0313	0.0027	0.0164	0.0020	0.0465	0.0034
A	8.1101	1.4794	6.9103	1.8306	8.6736	1.3260
μ	14.2728	0.3625	14.2329	0.5102	14.2752	0.3073
τ	0.1190	0.0020	0.1188	0.0027	0.1190	0.0018
**Output**	**(c) Input: *RR*(*Z*) = *exp*(*β*(*Z*)), *β*(*Z*) = *θ* × *log*(*1 + Z*/*A*) × *LF*(*Z*,*τ*,*μ*)**					
θ	0.0816	0.0047	0.0420	0.0025	0.1240	0.0073
A	50.9900	3.6630	39.6000	3.0700	57.8600	4.1180
μ	13.0700	0.0491	13.0100	0.0567	13.1000	0.0469
τ	0.0274	0.0009	0.0267	0.0010	0.0277	0.0008

**Table 3:** Relative risks (RR) estimated from the presented approaches at *Z* = O_3_ = 30 ppb.

Lags	Method	RR	95%CI	AIC Value
Lag 0	Table 1a	1.0564	1.0325, 1.0809	3.25140 × 10^4^
Lag 0	Table 1b	1.0530	1.0327, 1.0737	3.25099 × 10^4^
Lag 0	Table 1c	1.0522	1.0366, 1.0681	3.24914 × 10^4^
All lags	Table 2a	1.0531	1.0231, 1.0718	N/A
All lags	Table 2b	1.0462	1.0253, 1.0677	N/A
All lags	Table 2c	1.0387	1.0240, 1.0531	N/A

## Data Availability

The used health data are available at the location: (https://www.albertahealthservices.ca/zones/edmonton-zone.aspx) (accessed on 28 June 2022). The NAPS (environmental) data are at the location: http://maps-cartes.ec.gc.ca/rnspa-naps/data.aspx (accessed on 28 June 2022).
